# Coevolution of the Toll-Like Receptor 4 Complex with Calgranulins and Lipopolysaccharide

**DOI:** 10.3389/fimmu.2018.00304

**Published:** 2018-02-21

**Authors:** Andrea N. Loes, Jamie T. Bridgham, Michael J. Harms

**Affiliations:** ^1^Department of Chemistry and Biochemistry, University of Oregon, Eugene, OR, United States; ^2^Institute of Molecular Biology, University of Oregon, Eugene, OR, United States; ^3^Institute of Ecology and Evolution, University of Oregon, Eugene, OR, United States

**Keywords:** toll-like receptor 4, lipopolysaccharide, evolution, calgranulin, S100A9, MRP-126, marsupial, chicken

## Abstract

Toll-like receptor 4 (TLR4) induces inflammation in response to both pathogen- and host-derived molecules. Lipopolysaccharide (LPS) recognition by TLR4 has been shown to occur across the amniotes, but endogenous signaling through TLR4 has not been validated outside of placental mammals. To determine whether endogenous danger signaling is also shared across amniotes, we studied the evolution of TLR4-activation by the calgranulin proteins (S100A8, S100A9, and S100A12), a clade of host molecules that potently activate TLR4 in placental mammals. We performed phylogenetic and syntenic analysis and found MRP-126—a gene in birds and reptiles—is likely orthologous to the mammalian calgranulins. We then used an *ex vivo* TLR4 activation assay to establish that calgranulin pro-inflammatory activity is not specific to placental mammals, but is also exhibited by representative marsupial and sauropsid species. This activity is strongly dependent on the cofactors CD14 and MD-2 for all species studied, suggesting a conserved mode of activation across the amniotes. Ortholog complementation experiments between the calgranulins, TLR4, CD14, and MD-2 revealed extensive lineage specific-coevolution and multi-way interactions between components that are necessary for the activation of NF-κB signaling by calgranulins and LPS. Our work demonstrates that calgranulin activation of TLR4 evolved at least ~320 million years ago and has been conserved in the amniote innate immune system.

## Introduction

Calgranulin proteins—including S100A8, S100A9, and S100A12—potently activate inflammation *via* an interaction with Toll-like receptor 4 (TLR4) (1–8). These damage-associated molecular pattern (DAMP) proteins play important roles in wound healing and vascular development, but can also lead to upregulation and amplification of the inflammatory response in arthritis, atherosclerosis, and inflammatory bowel disease (9–15). S100A9 has been identified as a potential drug target for inhibiting inflammation *via* inhibition of this pathway (3, 16). The molecular basis of calgranulin activation of TLR4 is not well understood, but involves direct binding of the calgranulin to the TLR4/MD-2/CD14 complex in a calcium and zinc-dependent manner (3, 17).

Calgranulin activation of TLR4 is often compared to lipopolysaccharide (LPS) activation of TLR4 (2–4). LPS is a pathogen-associated molecular pattern (PAMP) found in the cell walls of Gram-negative bacteria. LPS activation of TLR4 is relatively well understood (18): CD14 binds to LPS and loads it on to an TLR4/MD-2 complex (19, 20). This induces a conformational change that leads to homodimerization of TLR4/MD-2 and triggers an NF-κB cascade (21, 22). In some ways, calgranulin activation is similar. S100A9 has been shown to directly interact with CD14 as well as the TLR4/MD-2 complex, possibly indicating similar mechanism of activation (3, 17). In other ways, however, it is quite different. Most notably, LPS is a small molecule that is enveloped by MD-2—an unlikely activation pathway for a calgranulin protein (19, 23–27). Furthermore, LPS and calgranulins induce distinct downstream inflammatory responses, suggesting different modes of action (8, 28).

A better understanding of the differences between LPS and calgranulin activation of TLR4 may reveal the unique requirements for calgranulin activation of TLR4. This could allow elucidation of the molecular mechanism of calgranulin activation. Furthermore, the ability to independently modulate PAMP and DAMP activity could allow suppression of pathological DAMP inflammation independently of the pathogen response.

An evolutionary lens provides a powerful means for dissecting these similarities and differences. Comparison of orthologs from different species is a natural way to reveal conserved—and presumably important—features of both DAMP and PAMP activation of TLR4. Additionally, studies of coevolution between the TLR4, MD-2, CD14, and calgranulins may reveal important species-specific interactions that, in turn, provide insight into the mode of activation for each agonist.

We set out to determine whether calgranulin activation of TLR4 was present outside of mammals. While LPS activation of TLR4 has been validated across amniotes (29–32), calgranulin activation has only been characterized in placental mammals (1, 3). Using a combination of phylogenetics and functional characterization of amniote orthologs, we establish that calgranulin activation of TLR4 evolved at least in the last common ancestor of all amniotes. While the basic mode of action is conserved, complementation experiments reveal extensive coevolution between TLR4 complex members over time. Crucially, coevolution of components in this complex has different effects on LPS and calgranulins, revealing different molecular requirements for these two agonists.

## Results

### Calgranulins are Found Across the Amniotes

**Figure 1:**
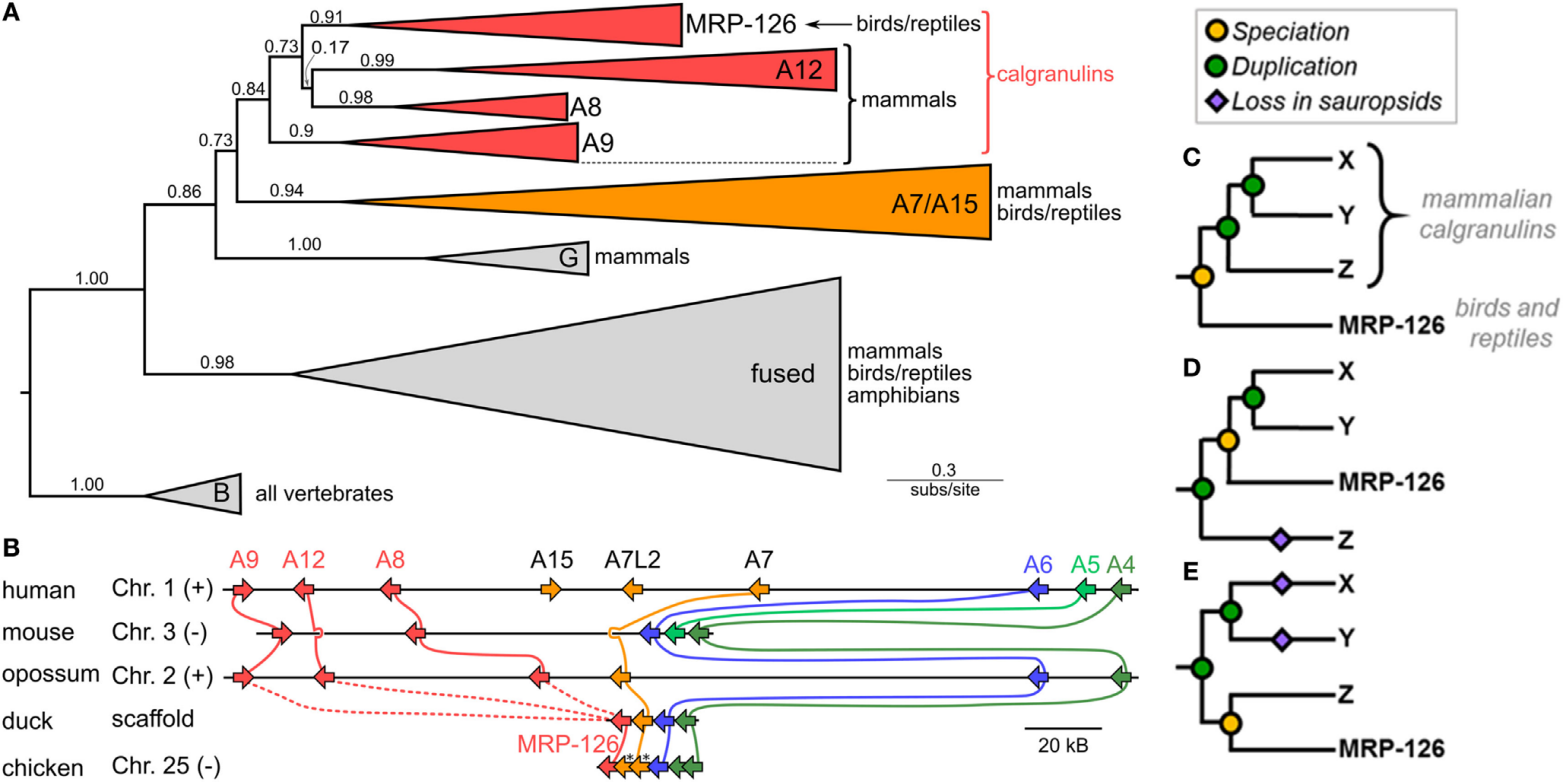
Phylogeny and synteny of the S100 family reveal a bird/reptile ortholog for mammalian calgranulin clade. **(A)** Maximum-likelihood phylogeny of 172 sequences from 30 taxa for proteins within a subclade of the S100 family. C-terminal tails of sequences were truncated prior to resolving the phylogeny. Wedges are collapsed clades of orthologs, with wedge height corresponding to the number of included taxa and wedge length indicating the longest branch length with the clade. Support values are SH-supports calculated using an approximate likelihood ratio test. Clades are colored to highlight calgranulins (red) or S100A7/A15 (orange). The taxa included in each clade are noted on the tree. **(B)** Genomic organization of S100 genes in amniote species. Arrows for genes represent the coding strand. Approximate distances between genes are represented by the length of line for selected chromosome. Mouse and chicken chromosomes were flipped to place A6 in the same orientation between species. Colors indicate orthology: calgranulins (red), A7/A15 (orange), A6 (blue), A5 (light green), and A4 (dark green). Solid lines between genomes indicate well-supported orthology from the phylogenetic tree. Dashed lines indicate ambiguous orthology. Sequences denoted with “*” were in the S100A7 clade in the maximum-likelihood tree shown in Figure S1B in Supplementary Material. **(C–E)** Possible relationships between MRP-126 and the mammalian calgranulins. Mammalian calgranulins are denoted as X, Y, and Z. (Because their branching is ambiguous, we cannot resolve which letter represents which mammalian calgranulin in these trees.) Icons indicate evolutionary events: speciation (yellow circle), duplication (green circle), or loss on sauropsid lineage (purple diamond). The panels are arranged from most parsimonious to least parsimonious. Note that, in the tree shown **(A)**, two marsupial proteins are grouped within the sauropsid MRP-126 clade. These are likely marsupial S100A12, which would be consistent with their location in the genome **(B)** and the lack of other marsupial S100A12 genes. In our more extensive analyses, the placement of these proteins is labile within the calgranulin clade (Figure S1 in Supplementary Material).

We first asked when the calgranulin proteins evolved. Calgranulins are members of the S100 protein family (33, 34). Previous phylogenies of the family allowed us to identify the close evolutionary relatives of mammalian calgranulins (33, 35, 36) but did not provide sufficient resolution to dissect the calgranulin clade itself. We constructed a curated multiple sequence alignment of 172 protein sequences from 30 species (Data Sheet S1 in Supplementary Material). All sequences were close homologs of the calgranulins, with the exception of S100B sequences included as an outgroup. We constructed a maximum-likelihood phylogeny for these proteins. This revealed eight well-supported clades corresponding to the paralog sequences we selected when we built the alignment (Figure 1A). The mammalian calgranulins (S100A8, S100A9, and S100A12) formed a clade with MRP-126, a protein found in birds and reptiles (sauropsids), but not mammals.

To validate the close relationship between these proteins, we examined the organization of these genes in the human, mouse, opossum, duck, and chicken genomes. We found that these genes exhibit synteny with one another, consistent with their placement together in the model-based phylogenetic analyses (Figure 1B). In humans, the genes encoding S100A8, S100A9, and S100A12 are near the 5′ end of a contiguous cluster of S100 genes, flanked on the 3′ end by S100A6 and S100A4 (37). MRP-126 exists in an identical arrangement in its genome, albeit on the opposite strand (Table S1 in Supplementary Material). We attempted to verify this arrangement held for reptiles, but we were unable to find reptile genomes with contigs of sufficient length in this region to confidently assign synteny.

This analysis establishes MRP-126 as a sauropsid calgranulin; however, relationships within the calgranulin/MRP-126 clade were ambiguous. To try to better resolve the relationship between the calgranulins, we repeated our phylogenetic analysis using both a Bayesian approach and a second maximum-likelihood analysis with a larger set of 510 sequences (Data Sheet S2 in Supplementary Material). In both cases, we obtained similar results: S100A7, S100A8, S100A9, S100A12, and MRP-126 form a clade, but these proteins could not be confidently placed relative to one another (Figure S1 in Supplementary Material). Additional S100 proteins are found in this region of the genome in sauropsids. Sequences from *Anolis carolinensis, Gallus gallus, Pogona vitticeps*, and *Gekko japonicus* are included in our large ML tree (Figure S1B in Supplementary Material), but could not be confidently annotated with phylogenetic analysis. These sequences could be the result of lineage-specific duplications, gene conversion, or represent highly diverged orthologs to another S100 such as S100A7.

There are three basic scenarios consistent with our data (shown schematically in Figures 1C–E). The most parsimonious would have MRP-126 co-orthologous to S100A8, S100A9, and S100A12 (Figure 1C). This requires two duplication events on the mammalian lineage after the divergence of mammals and sauropsids. Alternatively, MRP-126 could be orthologous to two of the mammalian proteins, requiring two duplication events and one loss (Figure 1D). The least parsimonious scenario would have MRP-126 co-orthologous to one mammalian calgranulin. This would require two duplications and two losses (Figure 1E). Alternate scenarios exist but require more duplications and losses. The first scenario seems most plausible, given its parsimony; however, our data are insufficient to fully resolve these relationships. Whatever the precise branching pattern within the calgranulin clade, our analysis supports classifying MRP-126 as a sauropsid calgranulin.

Finally, despite extensive BLAST queries, we were unable to locate any calgranulin orthologs in amphibians or bony fishes. Because of the large number of high quality fish genomes, this likely indicates that calgranulins are specific to tetrapods. There are relatively few amphibian genomes, so it remains unclear whether the lack for calgranulins in amphibans represents poor sampling or a true origin in the amniote ancestor.

### Calgranulin DAMP Activity Exists Across Amniotes

**Figure 2:**
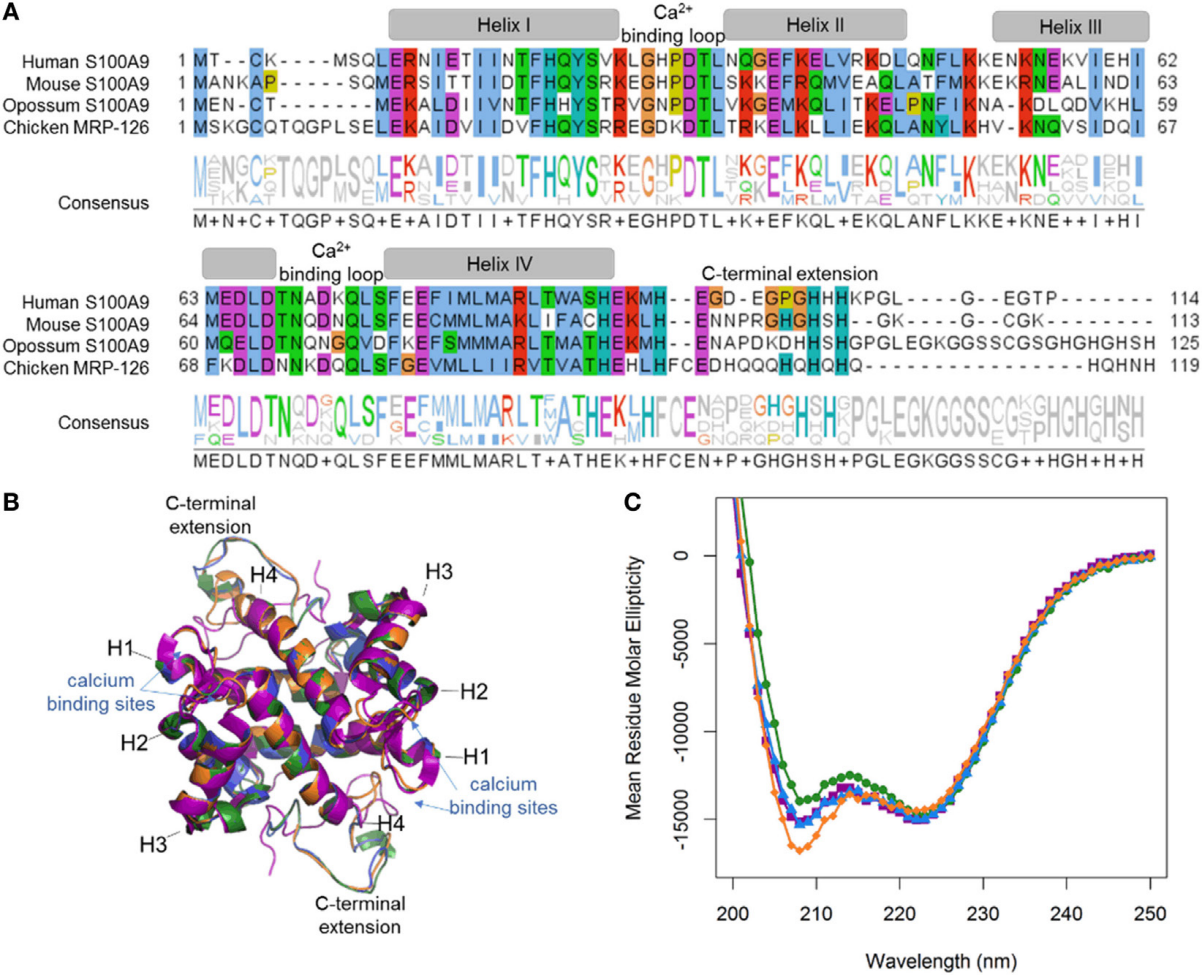
MRP-126, as well as mammalian calgranulins, is primarily alpha-helical with a disordered C-terminal extension. **(A)** Multiple sequence alignment of mammalian calgranulins with chicken MRP-126. Amino acid residues are colored to show sites with similar chemical properties. **(B)** Homology model of mouse S100A9 (green), opossum S100A9 (blue), and chicken MRP-126 (orange) aligned with human S100A9 (purple) calcium-bound NMR structure *PDB: 5i8n*. **(C)** Circular dichroism spectra of mammalian calgranulins and chicken MRP-126.

Our phylogenetic analyses reveal that calgranulins arose at least in the ancestor of amniotes. To determine when DAMP activity evolved, we “walked out” along the tree, recombinantly expressing and purifying calgranulin proteins from the human (*Homo sapiens*), mouse (*Mus musculus*), opossum (*Monodelphis domestica)* and chicken (*Gallus gallus*). For the mammals, we selected S100A9 proteins which, in the human and mouse, are known to be potent DAMPs. We expressed chicken MRP-126 as a representative sauropsid calgranulin (Figure 2A). After purification, we verified that all proteins were folded using far-UV circular dichroism spectroscopy. As expected for S100 proteins, all four proteins gave largely alpha-helical structures (Figure 2B). We also built homology models of mouse S100A9, opossum S100A9 and chicken MRP-126 using the human S100A9 template (Figure 2C). All these sequences could be readily accommodated in the S100 fold (38).

We next asked how well each calgranulin could activate *via* TLR4. We assayed the TLR4-dependent activation of the inflammatory NF-κB pathway using an *ex vivo* cell culture assay (39, 40). We transiently transfected plasmid constructs encoding TLR4 and its cofactors MD-2 and CD14 into HEK293T/17 cells. These cells do not natively express TLR4, MD-2, or CD14 so TLR4-dependent activation of NF-κB depends on the heterologous expression of the transfected TLR4 complex components. We used a luciferase gene under the control of an NF-κB promoter as a reporter. The NF-κB pathway is conserved across bony vertebrates (41), making mammalian cells useful as a common host for amniote TLRs.

**Figure 3:**
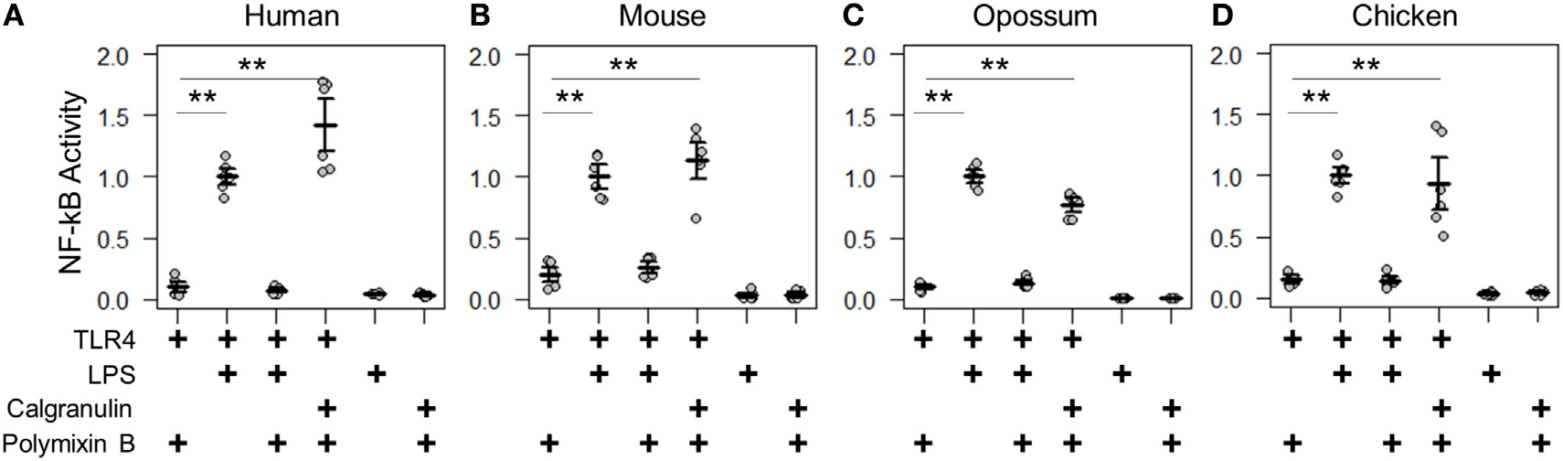
Calgranulin activation of toll-like receptor 4 (TLR4) is shared across amniotes. NF-κB activity for species-matched TLR4/MD-2/CD14 complexes in the presence of calgranulins from **(A)** human, **(B)** mouse, **(C)** opossum, and **(D)** chicken. A “+” in the table below each series indicates which components are included in that treatment. Lipopolysaccharide (LPS) is used as a positive control for expression and activation of the complex. Polymyxin B is included to control for endotoxin-mediated activation of the complex. Activity is normalized to LPS activity of positive control within each biological replicate (i.e., for human, each technical replicate is divided by the average LPS activation of hTLR4, hMD2, and hCD14 for that biological replicate). Points are the technical triplicates from three biological replicates. Bold lines are the mean of the biological replicates. Error bars are SEM of the biological replicates. A two-tailed *t*-test was used to assess significance of difference in mean between the indicated series (**p-value <0.01).

We first validated the assay by measuring the known PAMP and DAMP activation of TLR4 complexes by the human and mouse proteins (2–4). We transfected genes encoding TLR4, MD-2, and CD14 into cells and measured activity under different treatment conditions (Figures 3A,B). LPS induced significantly higher NF-κB response than a mock treatment. The addition of the LPS sequestering agent polymyxin B completely abolished this activity. We then treated transfected cells with recombinantly expressed S100A9—the most potent DAMP of the mammalian calgranulins. Both the human and mouse TLR4 complexes responded robustly to the addition of species-matched S100A9. All treatment buffers, with the exception of the LPS treatment, contained polymyxin B to eliminate spurious activation *via* potential LPS contamination. Both LPS and S100A9 activation were strictly dependent on the presence of heterologous TLR4 (Figures 3A,B).

With the assay validated, we then turned our attention to the TLR4 complex from other amniotes. We started by assessing a marsupial, the opossum. TLR4, MD-2, CD14, and S100A9 are known to be expressed in marsupials (42, 43), but their activity had not been characterized for LPS or DAMP activation. We transfected opossum TLR4 complex components and treated them with both LPS and recombinant opossum S100A9. We found an identical pattern of activation for the opossum proteins relative to those from human and mouse (Figure 3C).

We next assessed the activation of the chicken TLR4 complex by LPS and MRP-126. Chicken MRP-126 is known to have a similar expression profile to mammalian calgranulins, with upregulated expression during bacterial infection (44, 45), suggesting it could play a similar role in inflammation. Chicken CD14 had not previously been functionally characterized for a role in LPS activation (44, 46). We transfected TLR4 complex components from the chicken. We observed an identical pattern of activation for the chicken as for the mammalian proteins for activation of the chicken TLR4 complex by LPS and recombinant MRP-126 (Figure 3D).

### Calgranulins from Across the Amniotes Activate TLR4 in a Similar Fashion

**Figure 4:**
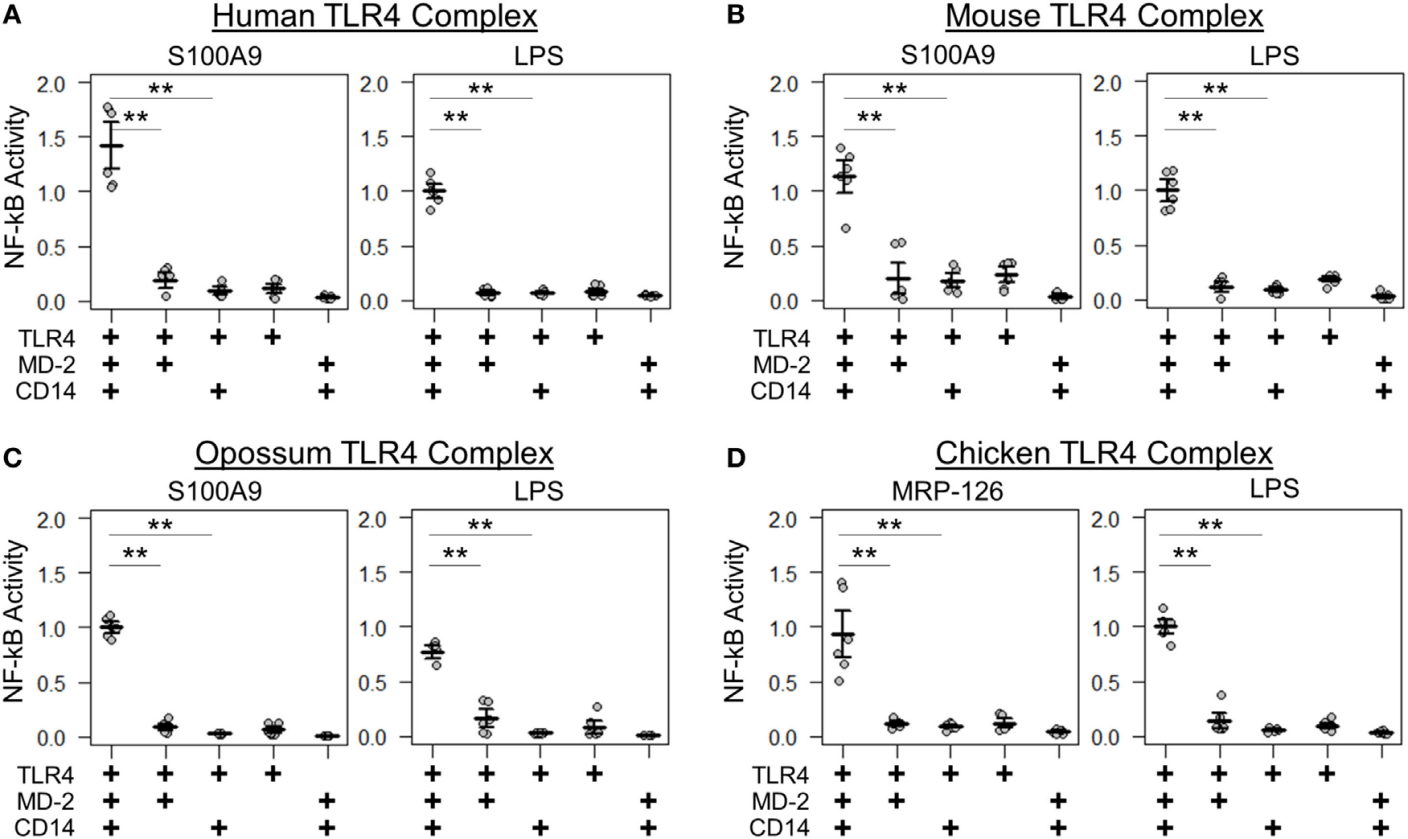
Amniote calgranulins have similar toll-like receptor 4 (TLR4) complex requirements. NF-κB activation of TLR4 from **(A)** human, **(B)** mouse, **(C)** opossum, and **(D)** chicken. NF-κB is normalized to lipopolysaccharide (LPS) activation of the control complex for that species. Points are the technical replicates from three biological replicates. Bold lines are the mean of the biological replicates. Error bars are SEM of the biological replicates. A “+” in the panel below indicates which components are included in the treatment. A two-tailed t-test was used to assess significance of difference in mean between the indicated series (**p-value <0.01).

We next determined whether the same components were necessary and sufficient for calgranulin activation of TLR4 across the amniotes. Shared complex requirements are strong evidence for a common, ancestral mode of action. We first measured the ability of mammalian S100A9 and chicken MRP-126 to activate their species-matched TLR4 with and without the cofactors MD-2 and CD14 present. We found that MD-2 was required for calgranulin activation of TLR4 in all species, as no MD-2-independent signaling was observed for any species tested (Figure 4). CD14 also strongly contributed to signaling. This is consistent with previous observations that CD14 is involved in TLR4-dependent NF-κB signaling by human S100A9 (17). Some CD14-independent NF-κB signaling was observed in the human and mouse proteins; however, the addition of CD14 drastically improved the signal for activation. No significant signaling was observed for the opossum or chicken proteins in the absence of CD14 (Figure 4).

**Figure 5:**
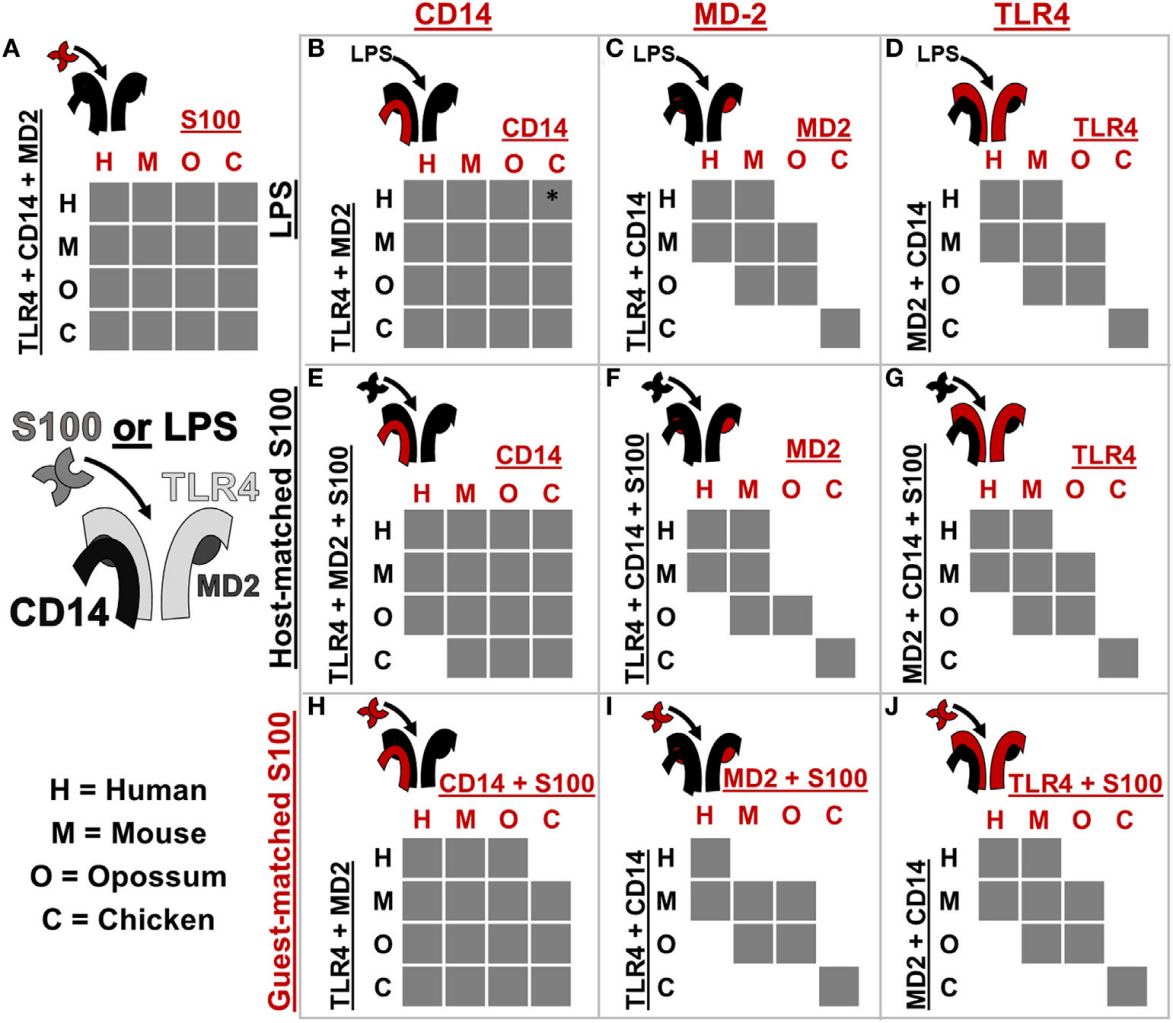
Toll-like receptor 4 (TLR4) complex components exhibit lineage-specific coevolution. **(A–J)** show activation of NF-κB signaling by receptor complexes assembled from host (black) and guest (red) proteins. Protein names on the left and top of each sub-panel indicate the components included. S100 is used to denote human S100A9, mouse S100A9, opossum S100A9, or chicken MRP-126. Letters indicate which species components come from: human (H), mouse (M), opossum (O), or chicken (C). If the cross-species complex activates >2-fold above the buffer control, the corresponding box is filled gray. The icon to the top-left of each sub-panel indicates graphically which components are being combined. *indicates > 2-fold activation above buffer control, but low total activation (see Figure S5 in Supplementary Material).

We next tested whether calgranulins from across the amniotes could complement one another. We treated the TLR4 complex from each species with recombinant calgranulin from each species and then measured activation. We found that all calgranulins could activate all complexes (Figure 5A), again supporting a shared ancestral mode of action. Dose–responsive activation occurs at concentrations in the micromolar range for all calgranulins against amniote TLR4/MD-2/CD14 complexes (Figure S2 in Supplementary Material).

### Calgranulins and LPS Signaling Have Overlapping, but Different Molecular Requirements

Coevolution can lead to species-specific interactions that, in turn, reveal key determinants of activation (26, 32). The cross-reactivity of calgranulins against TLR4 complexes from different species reveals a shared mode of action; however, we were also interested in identifying any species-specific differences in activation. We, therefore, searched for coevolution between the calgranulins and members of the TLR4 complex. We took a complementation approach, adding a guest component from one species into a host complex of components from a different species (Figure 5; Figures S3–S8 in Supplementary Material). We used this approach to test for lineage-specific evolution between TLR4, MD-2, CD14, and the calgranulins.

We first tested whether host–guest complexes were competent for LPS signaling. We used CD14 (Figure 5B), MD-2 (Figure 5C), and TLR4 (Figure 5D) as guests and then measured activation upon the addition of LPS. We found that CD14 was non-specific and could complement its orthologs from other species (Figure 5B). The lack of species-specificity for CD14 is consistent with it acting as a peripheral protein that delivers LPS to the central TLR4/MD-2 complex (47, 48).

By contrast, MD-2 and TLR4 exhibited strong species-specific variability in activation: many host–guest pairs were incompatible (Figures 5C,D). The only proteins that could complement one another fully were the mouse/human and mouse/opossum pairs. Furthermore, MD-2 and TLR4 gave essentially identical patterns of compatibility between species. This indicates strong coevolution between these two proteins. This likely arises as a result of the large, functionally critical interface formed between TLR4 and MD-2 (19, 21, 23, 26, 49–51).

We repeated these experiments using calgranulins rather than LPS to activate the complex. We used calgranulin matched to the host complex and added guest CD14 (Figure 5E), MD-2 (Figure 5F), and TLR4 (Figure 5G). This revealed a similar pattern to that of LPS signaling. CD14 was relatively non-specific (Figure 5E), while MD-2 and TLR4 were highly specific (Figures 5F,G). Indeed, the MD-2/TLR4 pattern of activation for calgranulins was identical to that of LPS, with the exception of the human-mouse and mouse-opossum heterocomplex. A similar pattern was observed when examining activation by the calgranulin matched to the guest component (Figures 5H–J).

**Figure 6:**
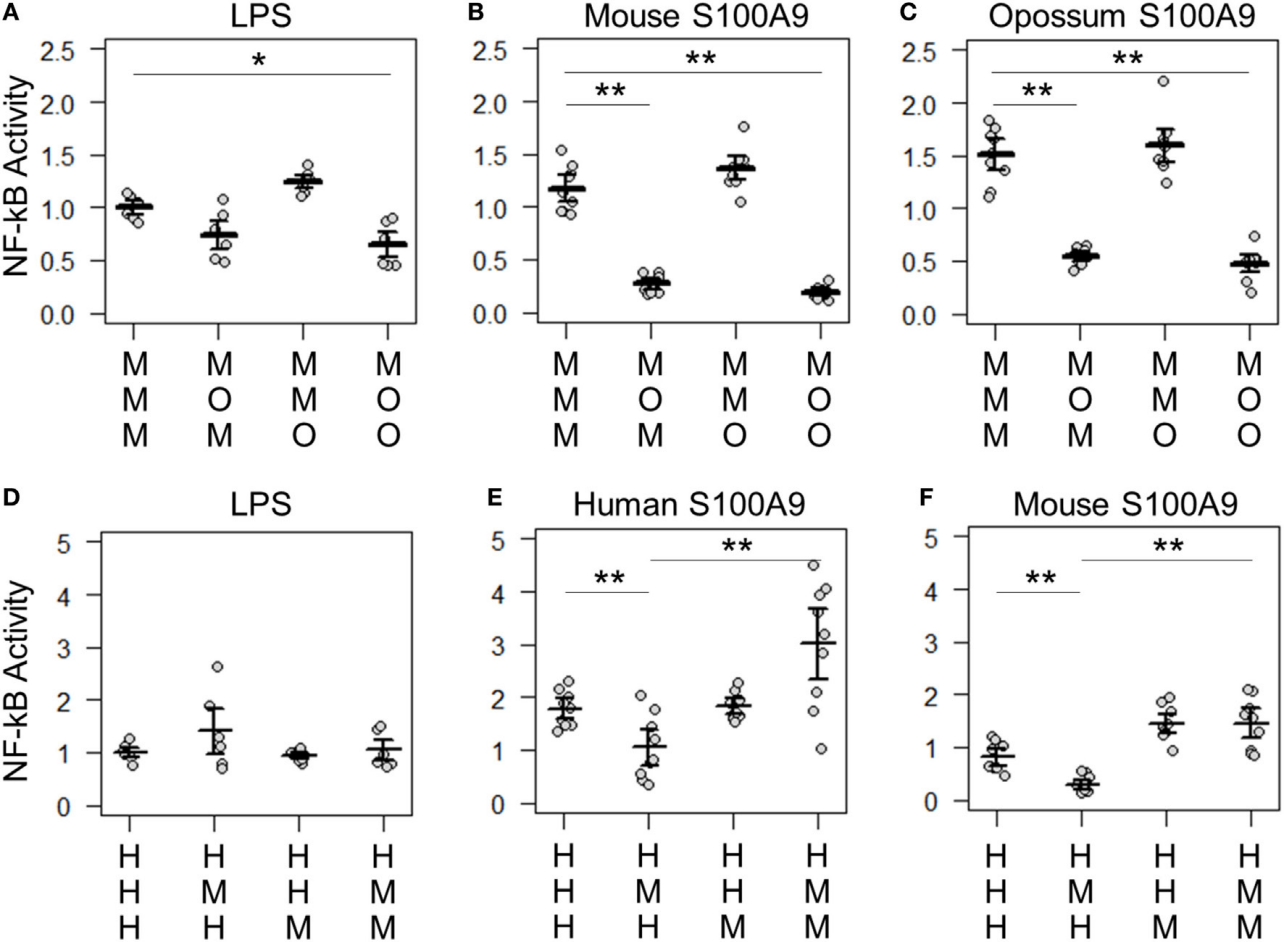
Lipopolysaccharide (LPS) and calgranulin exhibit different complex requirements. **(A–C)** show NF-κB activation of mouse toll-like receptor 4 (TLR4) with different combinations of mouse and opossum MD-2 and CD14. Activation by: **(A)** LPS, **(B)** mouse S100A9, and **(C)** opossum S100A9. **(D–F)** show NF-κB activation of human TLR4 with different combinations of human and mouse MD-2 and CD14. Activation by: **(D)** LPS, **(E)** human S100A9, and **(F)** mouse S100A9. Letters indicate which species the component was taken from: human (H), mouse (M), or opossum (O). NF-κB is normalized to LPS activation of the control complex for that species. Points are the technical triplicates from three biological replicates. Bold lines are the mean of the biological replicates. Error bars are SEM of the biological replicates. A two-tailed *t*-test was used to assess significance of difference in mean between the indicated series (*p-value <0.05, **p-value <0.01).

The host–guest combinations that do not activate with either LPS or calgranulin likely fail to assemble into a productive complex, independent of the nature of the pro-inflammatory signal. The failure of the mouse-opossum complexes for calgranulin but not LPS, however, indicates that there are different requirements to activate the complex *via* LPS or calgranulins. This can be seen with a more detailed comparison of the opossum/mouse MD-2 complementation analysis (Figures 6A–C). Opossum MD-2 can stand in for mouse MD-2 for LPS signaling (Figure 6A), but not for calgranulin signaling (Figures 6B,C). This reveals that these two pro-inflammatory signals activate differently, despite identical complex requirements (Figure 4).

There were also quantitative differences between LPS and calgranulin activation of human/mouse heterocomplexes. Mouse MD-2 fully complements human MD-2 for LPS activation of human TLR4 (Figure 6D). By contrast, mouse MD-2 is less efficient than human MD-2 for calgranulin activation of the complex (Figures 6E,F). Intriguingly, this difference can be offset by the addition of the mouse CD14 in addition to mouse MD-2 (Figures 6E,F). This interaction between MD-2 and CD14 reveals that both components are important for calgranulin activation of TLR4 and that their roles are different for LPS and calgranulin.

Our analysis reveals lineage-specific coevolution between complex members. The dominant signal for coevolution is between TLR4 and MD-2. This impacts both LPS and calgranulin activation, likely because the interaction between MD-2 and TLR4 is important for complex assembly. There are, however, differences between which host–guest complexes are sensitive to LPS and calgranulins, revealing that LPS and calgranulins activate these complexes in subtly different ways. These differences may indicate that the interface calgranulins interact with has independent molecular requirements from LPS, possibly spanning components or inducing an alternate active conformation than LPS.

## Discussion

Our work reveals that the calgranulin (DAMP) and LPS (PAMP) activation of TLR4 evolved at least as early as the ancestor of amniotes. As all amniote calgranulins we tested activate their species-matched TLR4, this activity likely arose before the divergence of amniotes and mammals ~320 million years ago. Like LPS, activation of NF-κB by calgranulins is strictly dependent on the cofactor MD-2 and potently increased by the cofactor CD14. These shared cofactor requirements suggest that both DAMP and PAMP use conserved, ancestral modes of activation.

We also find that DAMP and PAMP activation must occur *via* slightly different pathways. This can be seen in our complementation experiments. While opossum MD-2 can complement mouse MD-2 for LPS activation, it cannot complement mouse MD-2 for calgranulin activation. Likewise, mouse MD-2 requires mouse CD14 for calgranulin activation of human TLR4. These results could point to a direct interaction between MD-2 and S100A9 or a multi-way interaction between TLR4/MD-2/CD14 and calgranulins. Further analysis may help identify the residues involved in the TLR4/MD-2/calgranulin interface, as has been done for the MD-2/TLR4 interface by comparing human and other species activation by LPS (24, 32). Alternatively, this signal may be mediated indirectly, *via* the dimerization of TLR4/MD-2—activation of TLR4 requires dimerization of TLR4/MD-2, in part, mediated by residues on MD-2 (19, 22, 49, 52).

We found that calgranulins are broadly cross-reactive to TLR4 across the amniotes. This is consistent with a fairly non-specific mechanism of action, such as binding *via* a non-specific hydrophobic patch on the calgranulin surface. Hydrophobicity has been proposed to be an important global danger signal—exposure of hydrophobic regions of folded proteins is an indicator of damage (53). If a non-sequence-dependent mechanism of activation, such as hydrophobicity, is employed by the calgranulins for TLR4 activation, this may be a co-opted signaling mechanism which evolved prior to the calgranulins and is employed by other endogenous danger signals for TLRs.

It remains an open question when LPS activation evolved relative to calgranulin activation. One intriguing possibility is that the DAMP activation of TLR4 predates PAMP activation. TLR4 is expressed in bony fishes, but the ligand for this receptor is unknown. If another class of hydrophobic DAMP activates TLR4 in fish, DAMP activation of TLR4 may even predate amniotes. We do not yet know when LPS activation of TLR4 evolved. Resolving if DAMP activation evolved prior to PAMP activation will require studying TLR4 activation in amphibians and bony fishes.

Previous studies have investigated the ability of zebrafish TLR4 to activate NF-kB signaling in response to LPS (54–56). Because no orthologs to mammalian MD-2 and CD14 have been identified outside of amniotes, the researchers used mammalian CD14 and MD-2 in their experiments with zebrafish TLR4s. They observed no response to LPS. One interpretation of this result is that fish do not respond to LPS *via* TLR4. An observation that may support this is the loss of TLR4 in many fish lineages (54–56). Our complementation studies suggest that MD-2 coevolution with TLR4 would likely prohibit cross-reactivity of mammalian MD-2 with fish TLR4, even if fish possessed a functional MD-2-like cofactor. LPS activation of TLR4 in fishes should, thus, be re-examined. This will, however, require identification of TLR4 cofactors from fish—if they exist—rather than cofactors from tetrapods.

Our experiments and evolutionary analysis have revealed that calgranulin activation of TLR4 evolved at least 180 million years earlier than previously appreciated. These results show that DAMP recognition occurred through TLR4 at least in early amniotes. For many years, the predominant theory of the innate immune system was that its primary role was to discriminate “self” from “non-self.” In the 1990s, Matzinger proposed the danger hypothesis (57)—which describes the innate immune system as a general danger sensor, able to respond not just to pathogen molecules but also to endogenous signals indicative of damage. Our results are consistent with an ancient, general danger-sensing function for TLR4: at least across amniotes TLR4 plays a role in combating danger, not simply as a mechanism to distinguish “self” and “non-self.”

## Materials and Methods

### Phylogenetics and Genomic Analysis

We constructed a curated database of S100 homologs from a consistent set of species. We obtained amino acids sequences of S100 proteins from a subset of the amniote species in Ensembl version_87 (58). We obtained additional bird and reptile S100A7 and MRP-126 sequences using the human calgranulin paralogs to BLAST against the NCBI database. This yielded a set of 172 sequences from 30 taxa (Data Sheet S1 and S3 in Supplementary Material). We constructed our multiple sequence alignment using MSAPROBS (59), followed by manual editing in MEGA (60). We trimmed the alignment to remove highly variable (and, therefore, unalignable) C-terminal extensions, as well as the non-S100 domains of the fused S100 proteins. We used PHYML-SS (61, 62) with subtree pruning and re-grafting to construct the ML phylogeny. Pilot analyses revealed that the LG substitution model with eight rate categories and a floating gamma distribution parameter yielded the highest likelihood trees (63–65). An AIC test was used to control for overfitting (66). We rooted our trees using the divergence of S100B, an ancient S100 found across jawed vertebrates. These steps were repeated with a larger set of 510 sequences representing S100s within this clade of the S100 family (Data Sheet S2, S4, S5, and S6 in Supplementary Material).

We constructed our Bayesian tree using ExaBayes (67) using a single data partition, integrating over tree topologies, rates, and evolutionary models. We ran two replicate analyses, using three heated chains and a single cold chain per replicate. We ran for 5.6 million rounds, with a final average difference between replicates of 0.6%. We discarded the first 15% of the analysis as burn in and generated a 50% majority-rule consensus tree.

The syntenic analysis was done using the Ensembl synteny module (68) to map orthologs and homologs onto the chromosomes of species of interest (Table S1 in Supplementary Material). The genome assemblies were *Homo sapiens* (GRCh38.p10; GCA_000001405.25), *Mus musculus* (GRCm38.p5; GCA_000001635.7), *Monodelphis domestica* (monDom5; GCF_000002295.2), *Anas platyrhynchos* (BGI_duck_1.0; GCA_000355885.1), and *Gallus gallus* (Gallus_gallus-5.0; GCA_000002315.3). We attempted to include representative reptile genome; however, the S100 genes of interest were found on different contigs in the available reptile genomes.

### Plasmids and Recombinant Protein Preparation and Characterization

Mammalian expression vectors containing human TLR4 and mouse TLR4 were a gift from Ruslan Medzhitov (Addgene plasmid #13085 and #13086) and human CD14 and ELAM-Luc were a gift from Doug Golenbock (Addgene plasmid #13645 and #13029). Human MD-2 was obtained from the DNASU Repository (HsCD00439889). Mouse MD-2 (UniProt #Q9JHF9) and CD14 (UniProt #P10810), as well as opossum TLR4 (UniProt #F6Y6W8), MD-2 (UniProt #F6QBE6), CD14 (NCB Accession #XP_007473804.1) and chicken TLR4 (UniProt #C4PCF3), MD-2 (UniProt #A0A1D5NZX9), and CD14 (UniProt #B0BL87) were designed to be free of restriction sites and codon optimized for human expression and purchased as mammalian expression vector constructs in pcDNA3.1(+) from Genewiz (NJ, USA).

Synthetic gene constructs for human (UniProt #P06702), mouse (UniProt #P31725), and opossum (UniProt #F7AJJ0) S100A9 and chicken MRP-126 (UniProt #P28318) were also designed to be free of common restriction sites and codon optimized and purchased as PUC57 constructs from Genewiz (NJ, USA). Genes were cloned into a modified 6 × His MBP LIC TEV vector with NcoI and HindIII to yield protein constructs with TEV-cleavable histidine tags. *E. coli* BL21(DE3) pLysS competent cells containing the expression vectors for S100 proteins were grown overnight at 37°C, diluted1:150 into LB containing ampicillin and chloramphenicol, grown to mid-log phase (OD_600_ ~ 0.6–1), and induced with 1 mM isopropyl-β-d-1-thiogalactopyranoside overnight at 16°C with aeration. Bacterial pellets were harvested by centrifugation at 3,000 rpm at 4°C for 20 min and stored at −20°C. Pellets (~5 g) were suspended by vortexing and lysed in 25 mL Buffer (25 mM Tris, 100 mM NaCl, 25 mM imidazole, pH 7.4) with 37.5 U DNase I (ThermoFisher Scientific) and 0.75 mg Lysozyme (ThermoFisher Scientific) by shaking at RT for approximately 1 h. The lysate was clarified by centrifugation at 15,000 RPM for 50 min at 4°C. Protein was purified using a 5 mL Ni^2+^-NTA HisTrap column from (Healthcare GE) using an FPLC (Akta Biosciences) with gradient elution to HisB (25 mM Tris, 100 mM NaCl, pH 7.4, with 500 mM imidazole for human, mouse, and chicken proteins, 1 M imidazole for opossum S100A9). Pooled elution peak of purified protein was cleaved with tobacco etch protease (TEV) overnight at RT. Cleaved protein was collected from a gradient elution of a 5 mL Ni-NTA column from HisA to HisB. Protein purity was assessed with SDS-PAGE and pure fractions were pooled and dialyzed into phosphate buffered saline (PBS), 0.5 mM TCEP, pH 7.4. Protein was flash frozen in liquid nitrogen and fresh aliquots were thawed weekly for functional assays. TLR4 activation was tested with two independent preps of each protein to ensure that the results were not batch specific. Protein concentrations were measured using a Bradford assay.

Homology models of mouse S100A9, opossum S100A9, and chicken MRP-126 were prepared with Swiss-Model (69, 70). Alignment was constructed with Jalview (71). For secondary structure measurements, protein samples were prepared at 10 µM in endotoxin free PBS and far-UV circular dichroism data were collected between 200 and 250 nm using a J-815 CD spectrometer (Jasco) with a 1 mm quartz spectrophotometer cell (Starna Cells, Inc., Catalog No. 1-Q-1). Duplicate scans were collected for each protein and averaged. A blank buffer spectra was subtracted from sample measurements and raw ellipticity was converted into mean molar ellipticity using concentration and the number of residues for each protein.

### Cell Culture and Transfection Conditions

Human embryonic kidney cells (HEK293T/17, ATCC CRL-11268) were maintained up to 30 passages in DMEM supplemented with 10% FBS at 37°C with 5% CO_2_. For each transfection, a confluent 100 mm plate of HEK293T/17 cells was treated at room temperature with 0.25% Trypsin-EDTA in HBSS and resuspended with an addition of DMEM + 10% FBS. This was diluted fourfold into fresh medium and 135 µL aliquots of resuspended cells were transferred to a 96-well cell culture treated plate. Transfection mixes were made with 10 ng of TLR4, 1 ng of CD14, 0.5 ng of MD-2, 0.1 ng of Renilla, 20 ng of ELAM-Luc, and 68.4 ng pcDNA3.1(+) per well for a total of 100 ng of DNA, diluted in OptiMEM to a volume of 10 μL/well. To the DNA mix, 0.5 µL per well of PLUS reagent was added followed by a brief vortex and RT incubation for 10 min. Lipofectamine was diluted 0.5 µL into 9.5 µL OptiMEM per well. This was added to the DNA + PLUS mix, vortexed briefly, and incubated at RT for 15 min. The transfection mix was diluted to 65 μL/well in OptiMEM and aliquoted onto a plate. Cells were incubated with transfection mix overnight (18–22 h) and then treated with protein (2 µM) or LPS (100 ng/well) mixtures (prepared in 25% PBS, 75% DMEM). *E. coli* K-12 LPS (tlrl-eklps, Invivogen) was dissolved at 5 mg/mL in endotoxin free water, aliquots were stored at −20°C. To avoid freeze-thaw cycles, working stocks of LPS were prepared at 10μg/mL and stored at 4°C. There has been some concern in testing recombinant DAMPs against TLR4 due to the potential presence of contaminating LPS in proteins which have been expressed in bacteria (72, 73). We tested our S100s in the presence of 50 µg/mL polymyxin B, an LPS binding agent to limit signaling from LPS contamination in recombinant protein preparations. This concentration of polymyxin B, while sufficient to eliminate signaling by 100 ng/mL of LPS had a minimal effect on the signaling by calgranulins (Figure S9 in Supplementary Material). Cells were incubated with treatments for 4 hr. The Dual-Glo Luciferase Assay System (Promega) was used to assay Firefly and Renilla luciferase activity of individual wells. Each NF-κB induction value shown represents the Firefly luciferase activity/Renilla luciferase activity, normalized to the LPS-treated transfection control for each species in order to normalize between plates. It should be noted that the level of constitutive activity was different between TLR4s from different species (Figure S10 in Supplementary Material).

For cross-species comparisons, we used an internal standard to account for systematic differences in the magnitude of luciferase activation between biological replicates. We normalized measured activation values such that the LPS activation of the reference gene combination was 1.0. This is an appropriate normalization because we only compare the relative activation of gene/activator combinations on the same plate. For example, for each biological replicate plate used in the human/mouse complementation analysis, we divided the activation of all gene/activator combinations on that plate by the LPS activation hTLR4/hMD2/hCD14 observed for that plate. This means we are comparing the activation of other combinations (e.g., hA9 activation of hTLR4/mMD2/mCD14) relative to the internal standard (LPS activation hTLR4/hMD2/hCD14).

## Author Contributions

AL and MH designed the study and wrote the paper. AL designed, performed, and analyzed the experiments. JB provided technical expertise. All authors analyzed the results and approved the final version of the manuscript.

## Conflict of Interest Statement

The authors declare that the research was conducted in the absence of any commercial or financial relationships that could be construed as a potential conflict of interest.
