# Supplementary material for: Coevolution of the Toll-Like Receptor 4 Complex with Calgranulins and Lipopolysaccharide
Source: Front Immunol. 2018 Feb 21;9:304. doi: 10.3389/fimmu.2018.00304 (PMC5826337; doi:10.3389/fimmu.2018.00304)
Supplement: Supplementary file 1 [file Table_1.PDF]

Table S1. Genome locations for proteins shown for synteny analysis in human, mouse, opossum, duck and chicken. Genomic locations obtained from Ensembl database.

Organism	ID	Protein	Genomic Location
Homo sapiens	ENSG00000163220	S100A9	1:153357854-153361027
Homo sapiens	ENSG00000163221	S100A12	1:153373706-153375649
Homo sapiens	ENSG00000143546	S100A8	1:153390032-153391188
Homo sapiens	ENSG00000184330	S100A7A	1:153416524-153423225
Homo sapiens	ENSG00000197364	S100A7L2	1:153437058-153439949
Homo sapiens	ENSG00000143556	S100A7	1:153457744-153460701
Homo sapiens	ENSG00000197956	S100A6	1:153534599-153536244
Homo sapiens	ENSG00000196420	S100A5	1:153537147-153541765
Homo sapiens	ENSG00000196154	S100A4	1:153543613-153550136
Mus musculus	ENSMUSG00000056071	S100A9	3:90692632-90695721
Mus musculus	ENSMUSG00000056054	S100A8	3:90668978-90670035
Mus musculus	ENSMUSG00000001025	S100A6	3:90612882-90624181
Mus musculus	ENSMUSG00000001023	S100A5	3:90608523-90611780
Mus musculus	ENSMUSG00000001020	S100A4	3:90603771-90606045
Monodelphis domestica	ENSMODG00000017406	S100A9	2:187687578-187692267
Monodelphis domestica	ENSMODG00000017410	S100A12	2:187668840-187671749
Monodelphis domestica	ENSMODG00000017403	S100A8	2:187727543-187728740
Monodelphis domestica	ENSMODG00000017402	S100A7	2:187744792-187750533
Monodelphis domestica	ENSMODG00000017400	S100A6	2:187846539-187848831
Monodelphis domestica	ENSMODG00000017397	S100A4	2:187864578-187865727
Gallus gallus	ENSGALG00000024272	MRP-126	25:1885186-1886619
Gallus gallus	Unannotated	S100A7-like	25:1880201-1881658
Gallus gallus	Unannotated	S100A7	25:1877283-1877810
Gallus gallus	ENSGALG00000041826	S100A6	25:1874283-1875569
Gallus gallus	ENSGALG00000026613	S100A4-like	25:1873156-1873538
Gallus gallus	ENSGALG00000037599	S100A4	25:1868937-1871234
Anas platyrhynchos	ENSAPLG00000010189	MRP-126	KB742558.1: 42667-47205
Anas platyrhynchos	Unannotated	S100A7	KB742558: 39193-39333
Anas platyrhynchos	ENSAPLG00000010172	S100A6	KB742558.1: 32550-33337
Anas platyrhynchos	ENSAPLG00000010031	S100A4	KB742558.1: 30950-31363
