# Supplementary figures and images for: Coevolution of the Toll-Like Receptor 4 Complex with Calgranulins and Lipopolysaccharide
Source: Front Immunol. 2018 Feb 21;9:304. doi: 10.3389/fimmu.2018.00304 (PMC5826337; doi:10.3389/fimmu.2018.00304)

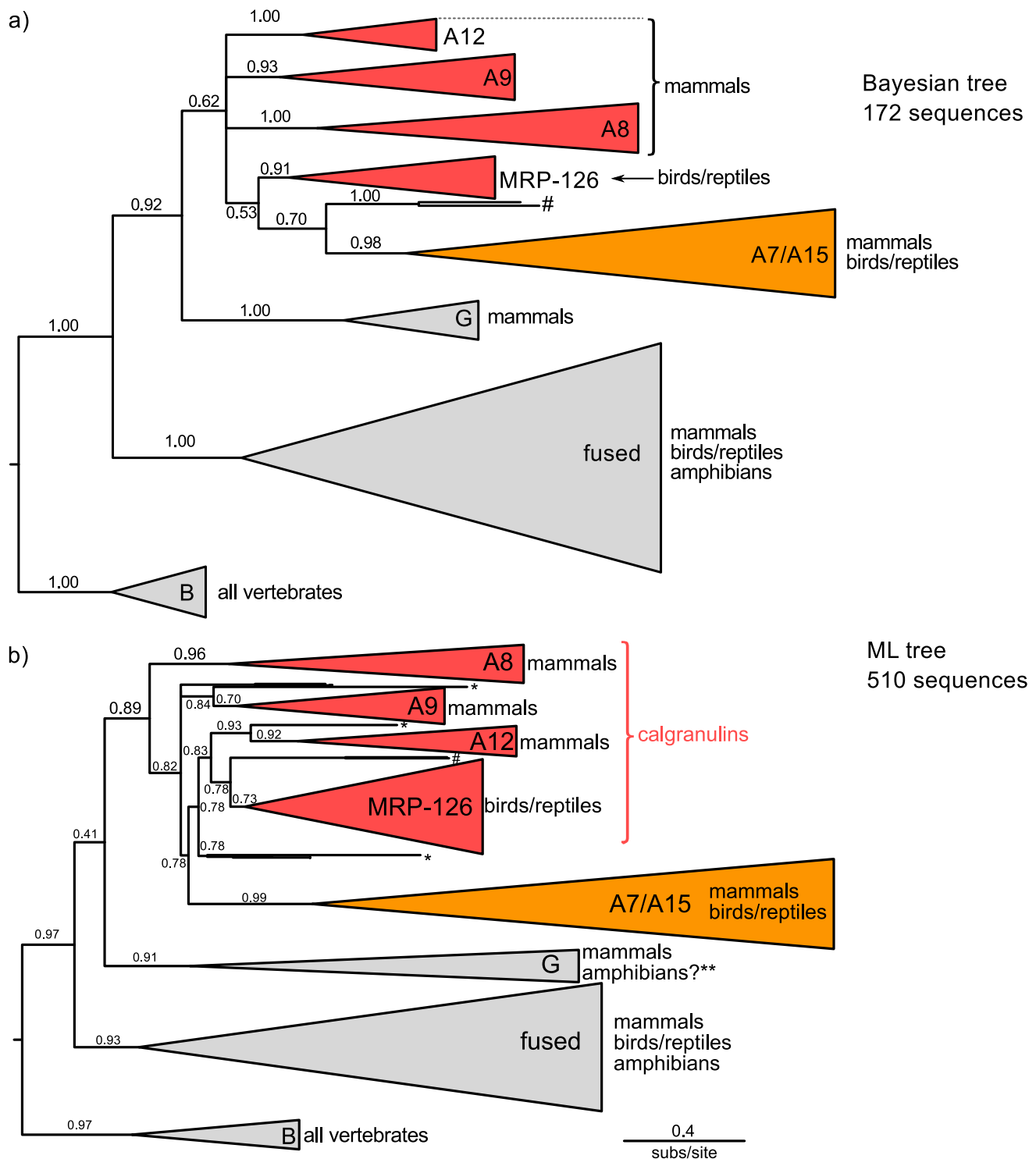

Supplement: Supplementary file 8 [file Image_1.PDF]
