# Supplementary material for: Coevolution of the Toll-Like Receptor 4 Complex with Calgranulins and Lipopolysaccharide
Source: Front Immunol. 2018 Feb 21;9:304. doi: 10.3389/fimmu.2018.00304 (PMC5826337; doi:10.3389/fimmu.2018.00304)
Supplement: Supplementary file 9 [file Image_2.PDF]

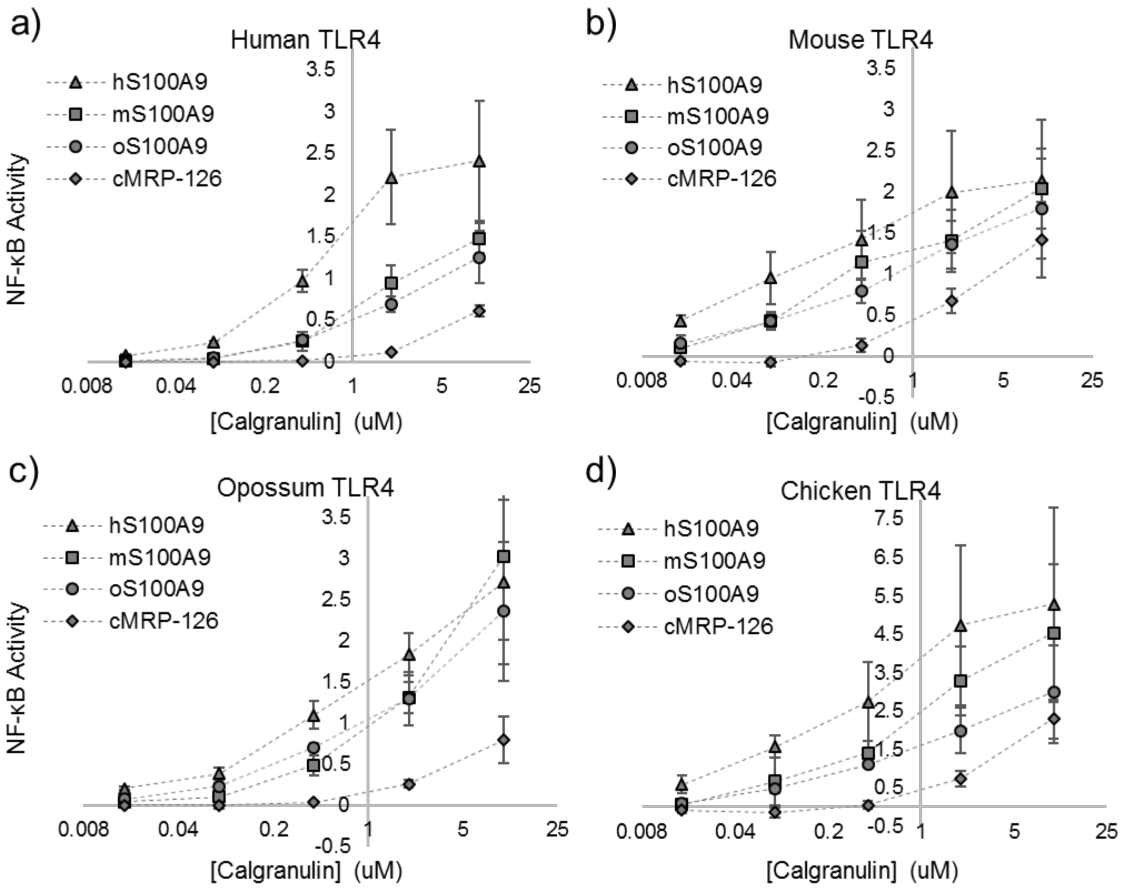

Figure S2. Dose response of calgranulins against alternate species TLR4/MD2/CD14 complexes NF-κB activity for calgranulins from human (▲), mouse (■), opossum (●), and chicken (◆) against TLR4/MD2/CD14 complexes from a) human, b) mouse, c) opossum and d) chicken. LPS was used as a positive control for expression and activation of the complex. Polymixin B is included to control for endotoxin-mediated activation of the complex. Activity is normalized to LPS activity of positive control within each biological replicate. Points are biological triplicates; box shows upper and lower quartiles; whiskers denote extremes for data series. Error bars for line plots are standard error of biological triplicates.
