# Supplementary material for: Coevolution of the Toll-Like Receptor 4 Complex with Calgranulins and Lipopolysaccharide
Source: Front Immunol. 2018 Feb 21;9:304. doi: 10.3389/fimmu.2018.00304 (PMC5826337; doi:10.3389/fimmu.2018.00304)
Supplement: Supplementary file 13 [file Image_6.PDF]

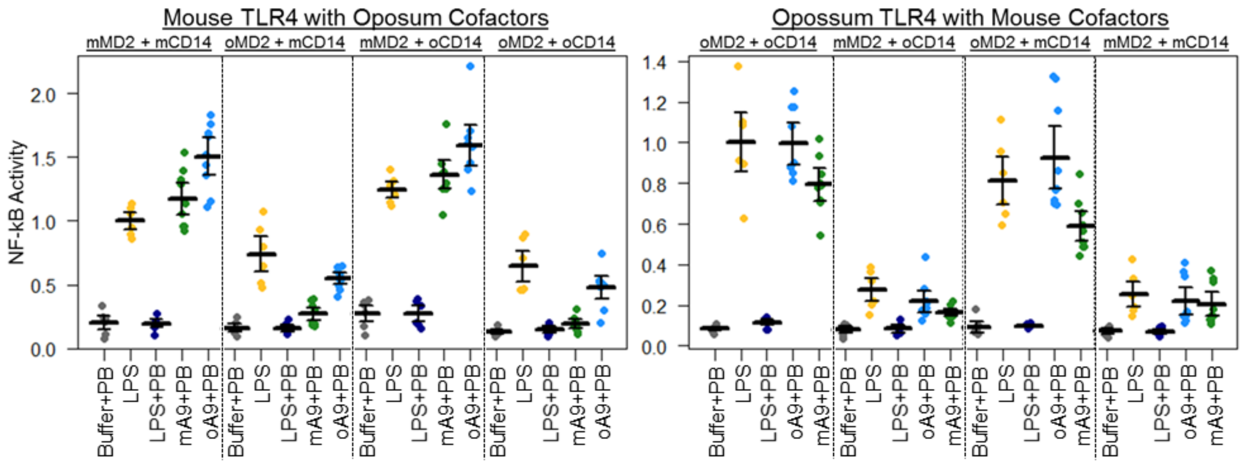

Figure S6. Complementation of TLR4 cofactors: mouse vs. opossum. Activation of mouse and opossum TLR4 in the presence of mouse and opossum MD-2 and CD14 by both LPS and calgranulins. Transfection conditions are noted above each panel. NF-κB is normalized to LPS activation of the control complex for that species. Points are the technical triplicates from three biological replicates; error bars show standard error with mean shown as a bold line. Colors show treatment conditions: Buffer + PB (grey), LPS (yellow), LPS+PB (navy), mouse S100A9 (green), opossum S100A9 (blue).
