# Supplementary material for: Coevolution of the Toll-Like Receptor 4 Complex with Calgranulins and Lipopolysaccharide
Source: Front Immunol. 2018 Feb 21;9:304. doi: 10.3389/fimmu.2018.00304 (PMC5826337; doi:10.3389/fimmu.2018.00304)
Supplement: Supplementary file 16 [file Image_9.PDF]

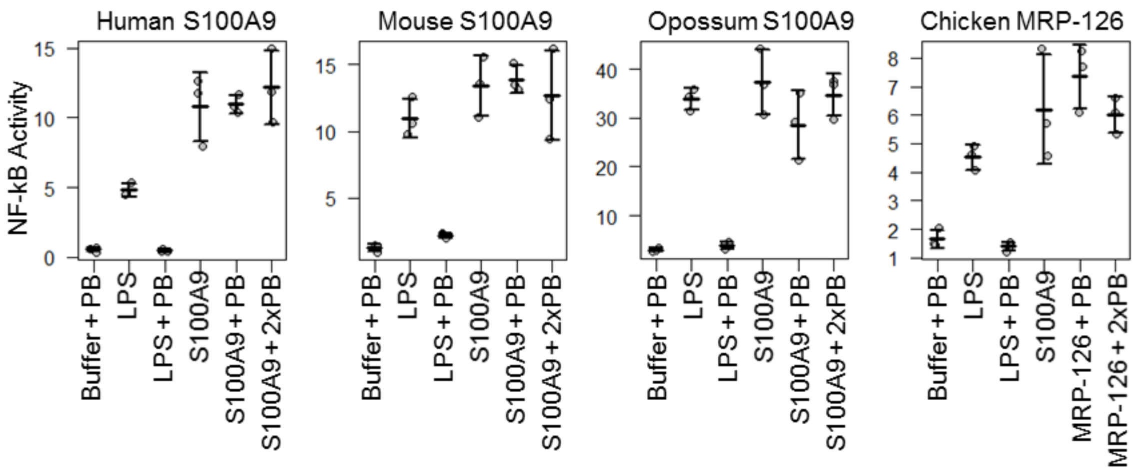

Figure S9. Activation of TLR4 by recombinant proteins in the presence and absence of polymixin B. NF-κB activity for TLR4/MD2/CD14 complexes from amniotes treated with LPS (100 ng/mL), or 2 uM recombinant protein in phosphate buffered saline with and without 50 ug/mL or 100 ug/mL Polymixin B (PB). Ratio of Firefly luciferase to Renilla luciferase is shown. Points are technical triplicates from a single biological replicate. Bold line shows mean of technical replicates. Error bars are standard deviation.
