# Supplementary material for: Coevolution of the Toll-Like Receptor 4 Complex with Calgranulins and Lipopolysaccharide
Source: Front Immunol. 2018 Feb 21;9:304. doi: 10.3389/fimmu.2018.00304 (PMC5826337; doi:10.3389/fimmu.2018.00304)
Supplement: Supplementary file 17 [file Image_10.PDF]

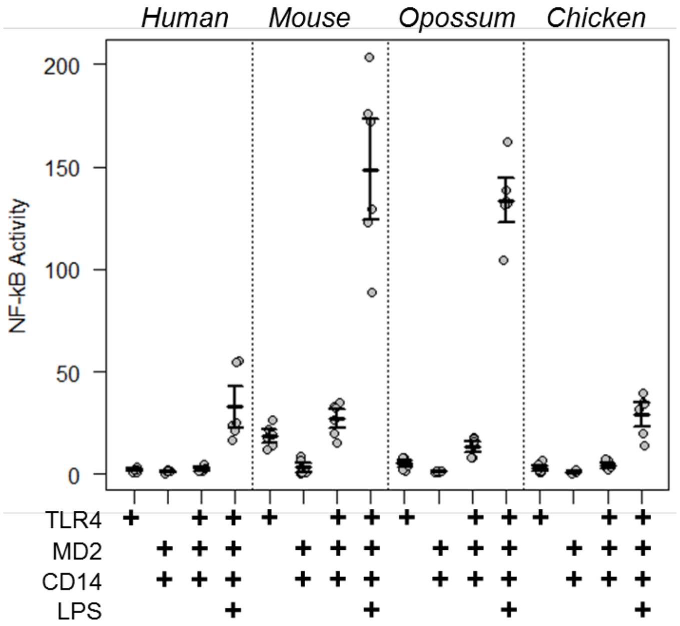

Figure S10. Constitutive activity of TLR4 complexes from different species NF-κB activity for TLR4/MD2/CD14 complexes from amniotes treated with phosphate buffered saline (PBS) with and without LPS (100 ng/mL). Polymixin B (50 ug/mL) was included in buffer samples to control for endotoxin-mediated activation of the complex. A "+" in the table below each series indicates which components are included in that treatment. Ratio of Firefly luciferase to Renilla luciferase is shown. Points are the technical replicates from three biological replicates; error bars show standard error with mean shown as a bold line, "+" in the panel below indicates which components are included in the treatment.
